# The prevalence and clinical features of *MYO7A*-related hearing loss including DFNA11, DFNB2 and USH1B

**DOI:** 10.1038/s41598-024-57415-1

**Published:** 2024-04-09

**Authors:** Kizuki Watanabe, Shin-ya Nishio, Shin-ichi Usami, the Deafness Gene Study Consortium, Takumi Kumai, Akihiro Katada, Noriko Ogasawara, Tomoko Shintani, Shin-ya Morita, Norito Takeichi, Shin-ichi Goto, Atsushi Nanba, Akira Sasaki, Yumiko Kobayashi, Yohei Honkura, Mika Adachi, Shunsuke Takai, Kiyoshi Oda, Teruyuki Sato, Takechiyo Yamada, Kazuhiro Shiina, Tsukasa Ito, Chikako Shinkawa, Akiko Amano, Daisuke Kikuchi, Hiroshi Ogawa, Tetsuro Wada, Yuki Hirose, Emiko Noguchi, Nobuko Moriyama, Kouji Ohtsuka, Kentaro Shirai, Rei Sadayasu, Mari Shimada, Hiroshi Suzumura, Tetsuya Tono, Masaomi Motegi, Ikko Mitoh, Hiroe Tada, Kyoko Nagai, Hideaki Sakata, Kotaro Ishikawa, Naohiro Yoshida, Kunio Mizutari, Yoichi Suzuki, Testuo Ikezono, Han Matsuda, Yoshihiro Noguchi, Hidehiko Takeda, Marina Kobayashi, Yuika Sakurai, Genki Hirabayashi, Shouri Tajima, Nobuhiro Nishiyama, Kyoko Shirai, Sachie Kawaguchi, Satoshi Iwasaki, Masahiro Takahashi, Sakiko Furutate, Shin-ichiro Oka, Hiroshi Yoshihashi, Hiroshi Futagawa, Naoki Ohishi, Makoto Hosoya, Yoshiyuki Kawashima, Taku Ito, Ayako Maruyama, Kozo Kumakawa, Takeshi Matsunobu, Naoko Sakuma, Katsutoshi Takahashi, Akinori Kashio, Hiroko Monobe, Yuji Miyoshi, Kumiko Yabuki, Yukiko Seto, Hajime Sano, Naomi Araki, Yasuhiro Arai, Mayuri Okami, Koichiro Wasano, Hiromitsu Hatakeyama, Yasuhiro Isono, Shinya Ohira, Manabu Komori, Shuji Izumi, Michiro Fujisaka, Atsushi Watanabe, Masayuki Okamoto, Yumi Ito, Mari Takahashi, Maiko Miyagawa, Yutaka Takumi, Hidekane Yoshimura, Jun Shinagawa, Hideaki Moteki, Koji Tsukamoto, Aya Ichinose, Natsuko Obara, Bunya Kuza, Natsuki Takada, Michinori Funato, Hiroshi Nakanishi, Shin-ichi Sano, Noriko Sano, Hiromi Koizumi, Tomoko Esaki, Tadao Yoshida, Satofumi Sugimoto, Takayuki Okano, Kazuhiko Takeuchi, Hiroshi Sakaida, Jun Nakayama, Masako Nakai, Risa Tona, Hiroshi Yamazaki, Mirei Taniguchi, Misako Hyogo, Takashi Nakamura, Mayumi Suematsu, Hiroaki Sato, Rie Horie, Hiroshi Hidaka, Akitoshi Mitani, Shin-ichi Haginomori, Akiko Ozaki, Yumi Ohta, Takaaki Minamidani, Shin-ichiro Kitajiri, Rie Kanai, Yasuyuki Hiratsuka, Naoki Yoshida, Chiho Okada, Natsumi Uehara, Yasushi Naito, Norio Yamamoto, Chihiro Morimoto, Mariko Kakudo, Muneki Hotomu, Masamitsu Kono, Yoshihiro Maegaki, Hiroyuki Awano, Tetsuya Okazaki, Tatsunori Sakamoto, Yuko Kataoka, Yukihide Maeda, Akiko Sugaya, Shin Masuda, Yukio Takeno, Takeshi Ishino, Kentaro Egusa, Yuji Yamashita, Kazuma Sugahara, Eiji Kondo, Naohito Hato, Masato Teraoka, Taisuke Kobayashi, Takashi Nakagawa, Nozomu Matumoto, Nana Tsuchihashi, Chie Oshikawa, Keiichiro Tsumadori, Kazuko Murakami, Ryota Mihashi, Eriko Shimazaki, Chiharu Kihara, Yukihiko Kanda, Kidzu YuUmi, Nanae Kawano, Kuniyuki Takahashi, Takeshi Nakamura, Toshiko Yuji, Ikuyo Miyanohara, Mikio Suzuki, Shunsuke Kondo

**Affiliations:** 1 Department of Otorhinolaryngology, Shinshu University School of Medicine, 3-1-1 Asahi, Matsumoto, Japan; 2 Department of Hearing Implant Sciences, Shinshu University School of Medicine, 3-1-1 Asahi, Matsumoto, 390-8621 Japan; 3 Asahikawa Medical University, Asahikawa, Japan; 4 Sapporo Medical University, Sapporo, Japan; 5 Tommo ENT Clinic, Sapporo, Japan; 6 Hokkaido University Graduate School of Medicine, Sapporo, Japan; 7 Shinoro ENT Clinic, Sapporo, Japan; 8 Hirosaki University Graduate School of Medicine, Hirosaki, Japan; 9 Nanba ENT Clinic, Hirosaki, Japan; 10 Aomori City Hospital, Aomori, Japan; 11 Iwate Medical University, Morioka, Japan; 12 Iwate Medical University Uchimaru Medical Center, Morioka, Japan; 13 Tohoku University Graduate School of Medicine, Sendai, Japan; 14 Tohoku Rousai Hospital, Sendai, Japan; 15 Tohoku Medical and Pharmaceutical University, Sendai, Japan; 16 Akita University Graduate School of Medicine, Akita, Japan; 17 Yamagata University School of Medicine, Yamagata, Japan; 18 Fukushima Medical University, Fukushima, Japan; 19 Fukushima Medical University Aizu Medical Center, Aizu, Japan; 20 University of Tsukuba, Tsukuba, Japan; 21 Hitachinaka General Hospital, Hitachi, Japan; 22 Tokyo Medical University Ibaraki Medical Center, Ami, Japan; 23 Tsuchiura Kyodo General Hospital, Tsuchiura, Japan; 24 Funaishikawa Skin and ENT Clinic, Tokai, Japan; 25 Jichi Medical University, Shimotsuke, Japan; 26 Dokkyo Medical University, Mibu, Japan; 27 International University of Health and Welfare Hospital, Nasushiobara, Japan; 28 Gunma University Graduate School of Medicine, Maebashi, Japan; 29 Takasaki ENT Clinic, Takasaki, Japan; 30 Kawagoe Otology Institute, Kawagoe, Japan; 31 National Rehabilitation Center for Persons with Disabilities, Tokorozawa, Japan; 32 Jichi Medical University Saitama Medical Center, Saitama, Japan; 33 National Defense Medical College, Tokorozawa, Japan; 34 Ageo Central General Hospital, Ageo, Japan; 35 Saitama Medical University, Moroyama, Japan; 36 International University of Health and Welfare School of Medicine, Narita, Japan; 37 Toranomon Hospital, Toranomon, Japan; 38 Jikei University School of Medicine, Nishishinbashi, Japan; 39 Juntendo University School of Medicine, Hongo, Japan; 40 Tokyo Medical University, Shinjyuku, Japan; 41 International University of Health and Welfare Mita Hospital, Mita, Japan; 42 Tokyo Metropolitan Children’s Medical Center, Fuchu, Japan; 43 Keio University School of Medicine, Shinjyuku, Japan; 44 Tokyo Medical and Dental University, Yushima, Japan; 45 Akasaka Toranomon Clinic, Akasaka, Japan; 46 Kamio Memorial Hospital, Kanda, Japan; 47 Nippon Medical School, Sendagi, Japan; 48 Showa General Hospital, Kodaira, Japan; 49 The University of Tokyo Faculty of Medicine, Hongo, Japan; 50 Japan Red Cross Medical Center, Hiroo, Japan; 51 Tokyo Metropolitan Tama Medical Center, Fuchu, Japan; 52 Toho University Faculty of Medicine, Ohmori, Japan; 53 Kitasato University School of Allied Health Sciences, Sagamihara, Japan; 54 Yokohama City University School of Medicine, Yokohama, Japan; 55 Tokai University School of Medicine, Isehara, Japan; 56 Yokohama City University Medical Center, Yokohama, Japan; 57 Shonan Kamakura General Hospital, Kamakura, Japan; 58 St. Marianna University School of Medicine, Kawasaki, Japan; 59 Niigata University School of Medicine, Niigata, Japan; 60 University of Toyama Faculty of Medicine, Toyama, Japan; 61 Graduate School of Medical Sciences, Kanazawa University, Kanazawa, Japan; 62 Fukui University School of Medical Sciences, Eiheiji, Japan; 63 University of Yamanashi Faculty of Medicine, Chuo, Japan; 64 Miyagawa ENT Hearing Clinic, Nagano, Japan; 65 Shinshu University School of Medicine, Matsumoto, Japan; 66 Aizawa Hospital, Matsumoto, Japan; 67 Iida Municipal Hospital, Iida, Japan; 68 Gifu University School of Medicine, Gifu, Japan; 69 Central Japan International Medical Center, Minokamo, Japan; 70 Gifu Prefectural General Medical Center, Gifu, Japan; 71 Nagara Medical Center, Gifu, Japan; 72 Hamamatsu University School of Medicine, Hamamatsu, Japan; 73 Kyoai Hospital, Fujinomiya, Japan; 74 Aichi Children’s Health and Medical Center, Oobu, Japan; 75 Nagoya University Graduate School of Medicine, Nagoya, Japan; 76 Fujita Health University, Toyoake, Japan; 77 Fujita Health University Bantane Hospital, Nagoya, Japan; 78 Mie University Graduate School of Medicine, Tsu, Japan; 79 Shiga University of Medical Science, Ootsu, Japan; 80 Shiga Medical Center for Children, Moriyama, Japan; 81 Kyoto University Graduate School of Medicine, Kyoto, Japan; 82 Kyoto Prefectural University of Medicine, Kyoto, Japan; 83 ENT Hyogo Clinic, Kyoto, Japan; 84 Kyoto Min-Iren Chuo Hospital, Kyoto, Japan; 85 Kansai Medical University, Hirakata, Japan; 86 Osaka Medical and Pharmaceutical University, Takatsuki, Japan; 87 Osaka University Graduate School of Medicine, Suita, Japan; 88 Izumi Clinic, Hannan, Japan; 89 Izumi Hearing Clinic, Hannan, Japan; 90 Kitajiri ENT Clinicl, Sakai, Japan; 91 Kitano Hospital, Osaka, Japan; 92 Osaka Red Cross Hospital, Osaka, Japan; 93 Hyogo Medical University, Nishinomiya, Japan; 94 Kobe University School of Medicine, Kobe, Japan; 95 Kobe City Medical Center General Hospital, Kobe, Japan; 96 Nara Medical University, Kashihara, Japan; 97 Kouseikai Takai Hospital, Tenri, Japan; 98 Wakayama Medical University, Wakayama, Japan; 99 Tottori University Faculty of Medicine, Yonago, Japan; 100 Shimane University Faculty of Medicine, Izumo, Japan; 101 Okayama University Graduate School of Medicine, Okayama, Japan; 102 Hiroshima Prefectural Hospital, Hiroshima, Japan; 103 Hiroshima University School of Medicine, Hiroshima, Japan; 104 Hiroshima City Hiroshima Citizens Hospital, Hiroshima, Japan; 105 Yamaguchi University Graduate School of Medicine, Ube, Japan; 106 Tokushima University Faculty of Medicine, Tokushima, Japan; 107 Ehime University School of Medicine, Toon, Japan; 108 Kochi Medical School, Nangoku, Japan; 109 Kyusyu University Graduate School of Medical Sciences, Fukuoka, Japan; 110 Fukuoka University Faculty of Medicine, Fukuoka, Japan; 111 Fukuoka Children’s Hospital, Fukuoka, Japan; 112 Kurume University School of Medicine, Kurume, Japan; 113 Saga University Faculty of Medicine, Saga, Japan; 114 Nagasaki University School of Medicine, Nagasaki, Japan; 115 Kanda ENT Clinic, Nagasaki, Japan; 116 Oita University Faculty of Medicine, Yufu, Japan; 117 Miyazaki University Faculty of Medicine, Miyazaki, Japan; 118 Kagoshima University Graduate School of Medicine, Kagoshima, Japan; 119 University of the Ryukyus Faculty of Medicine, Nishihara, Japan

**Keywords:** Genetics, Clinical genetics, Disease genetics

## Abstract

The *MYO7A* gene is known to be responsible for both syndromic hearing loss (Usher syndrome type1B:USH1B) and non-syndromic hearing loss including autosomal dominant and autosomal recessive inheritance (DFNA11, DFNB2). However, the prevalence and detailed clinical features of *MYO7A*-associated hearing loss across a large population remain unclear. In this study, we conducted next-generation sequencing analysis for a large cohort of 10,042 Japanese hearing loss patients. As a result, 137 patients were identified with *MYO7A*-associated hearing loss so that the prevalence among Japanese hearing loss patients was 1.36%. We identified 70 disease-causing candidate variants in this study, with 36 of them being novel variants. All variants identified in autosomal dominant cases were missense or in-frame deletion variants. Among the autosomal recessive cases, all patients had at least one missense variant. On the other hand, in patients with Usher syndrome, almost half of the patients carried biallelic null variants (nonsense, splicing, and frameshift variants). Most of the autosomal dominant cases showed late-onset progressive hearing loss. On the other hand, cases with autosomal recessive inheritance or Usher syndrome showed congenital or early-onset hearing loss. The visual symptoms in the Usher syndrome cases developed between age 5–15, and the condition was diagnosed at about 6–15 years of age.

## Introduction

One of the most common sensory disorders is hearing loss (HL), affecting one in 500–600 newborns^1^, and about 60% of cases of congenital HL are attributable to genetic causes^1^. Currently, more than 120 genes are known as genetic causes of sensorineural hearing loss^2^. With regard to genetic hearing loss, 70% of cases present with non-syndromic hearing loss, and 30% of cases present with syndromic hearing loss and demonstrate several symptoms associated with HL. Among the non-syndromic HL cases, 75–80% cases are categorized as autosomal recessive (AR) inheritance and about 20% cases are categorized as autosomal dominant (AD) inheritance^3^. The clinical features of HL patients, including the age at onset, progressiveness of HL, severity of HL, audiometric configuration, and the effectiveness of interventions, differ with causative gene and variant. Thus, genetic testing to identify the causative genes will be useful in enabling appropriate interventions for each individual patient.

The *MYO7A* gene was first reported as a causative gene for Usher syndrome by Weil et al., in 1995^4^. *MYO7A*, located on chromosome 11q13.5, consists of 49 exons which encode unconventional myosin (myosin 7a). Myosin 7a is expressed in the retina, lungs, testis, kidneys, and outer and inner hair cells in the inner ear^5^. In the inner ear, Myosin 7a forms a tripartite complex with SANS and Harmonin, and plays a crucial role in mechano-electro transduction in stereocilia, helps to maintain the mechanical tension across cadherin links and transports proteins components to the tip of stereocilia^6,7^. It is essential to the maintenance of hair cell stereocilia bundles and loss of this function is known to cause disorganized stereocilia and HL.

The *MYO7A* gene is responsible for autosomal dominant non-syndromic hearing loss (ADNSHL, locus DFNA11)^8^, and autosomal recessive non-syndromic hearing loss (ARNSHL, locus DFNB2)^9,10^. This gene is also known to be the most common genetic cause for Usher syndrome type 1 (USH1B), which is characterized by congenital severe-profound bilateral sensorineural hearing loss (SNHL), prepubertal onset retinitis pigmentosa (RP), and vestibular dysfunction^4^.

To date, 882 variants have been reported for *MYO7A*-associated HL. Most of the reported variants were identified as genetic causes for Usher syndrome, with only 35 variants reported as causative for ADNSHL and only 49 variants reported as causative for ARNSHL^11^. Only a limited number of reports on ADNSHL and ARNSHL patients are available; thus, the detailed clinical features of ADNSHL and ARNSHL patients remain unclear. In addition, most of the previous papers report on only a few cases or only Usher syndrome cases, and the prevalence of ADNSHL, ARNSHL and USH1B in large HL cohorts is also unclear.

In this study, we reported (1) the prevalence of *MYO7A*-associated HL for each of ADNSHL, ARNSHL, and USH1B in a large Japanese HL cohort, (2) the detailed clinical characteristics of each set of patients including the onset age, severity of HL, progressiveness of HL, and other associated symptoms (tinnitus, vertigo, visual symptoms), and (3) genotype–phenotype correlations for variant type and clinical phenotype.

## Results

### Identified variants and prevalence of *MYO7A*-associated hearing loss in a large Japanese hearing loss cohort

As shown in Tables 1 and 2, we identified 70 disease-causing candidate *MYO7A* variants. Among the 70 variants, 36 were novel variants and 34 were previously reported. Twenty-three of the identified variants were found in ADNSHL patients, with the remaining variants found in ARNSHL or USH1B patients.

**Table 1:** *MYO7A* candidate variants for DFNA11 identified in this study.

Exon	Base change	AA Change	Domain	SIFT	PP2HV	MutTaster	MutAssessor	REVEL	CADD Phred	dbscSNV	TOMMO 38KJPN	gnomAD exome	Pathogenicity	References
5	c.[420C>G;5503G>C]	p.[N140K;E1835Q]	Myosin head	D	P	D	L	0.415	20.3	−	0	0	Uncertain_Significance	This study
40			MyTH4 domain	T	B	D	L	0.593	13.9	−	0	0		
5	c.[439C>T;1436T>C]	p.[R147C;L479P]	Myosin head	D	D	D	H	0.67	34.0	−	0	0.000008	Uncertain_Significance	This study
13			Myosin head	T	P	D	N	0.468	23.7	−	0	0		
6	c.[479C>G;2947G>T]	p.[S160C;D983Y]	Myosin head	D	D	D	H	0.927	25.7	−	0	0	AD_Pathogenic	Iwasa YI et al., PLoS One. 2016;11:e016
24			–	D	B	D	M	0.573	28.2	−	0.000013	0		
6	c.547T>C	p.S183P	Myosin head	D	D	D	M	0.858	26.5	−	0	0	Uncertain_Significance	This study
7	c.689C>T	p.A230V	Myosin head	D	D	D	M	0.818	35.0	−	0	0	AD_Pathogenic	Di Leva F et al., Audiol Neurootol. 2006;11:157–64
13	c.1436T>C	p.L479P	Myosin head	T	P	D	N	0.468	23.7	−	0	0	AD_Pathogenic	This study
17	c.1966C>G	p.L656V	Myosin head	D	D	D	M	0.788	26.8	−	0.000039	0	Uncertain_Significance	This study
17	c.1978G>A	p.G660R	Myosin head	D	D	D	H	0.976	32.0	−	0	0	AD_Pathogenic	Iwasa YI et al,. PLoS One. 2016;11:e016
17	c.2003G>A	p.R668H	Myosin head	D	D	D	H	0.886	34.0	−	0	0	AD_Likely_Pathogenic	Sang Q et al., PLoS One. 2013;8:e55178
18	c.2185A>G	p.K729E	Myosin head	D	P	D	H	0.926	24.5	0.0662	0	0	Uncertain_Significance	This study
21	c.2558G>A	p.R853H	IQ motif	D	D	D	M	0.741	34.0	−	0	0	AD_Likely_Pathogenic	Shearer AE et al., J Med Genet. 2013;50(9):627–34
21	c.2558G>T	p.R853L	IQ motif	D	P	D	H	0.619	34.0	−	0	0	Uncertain_Significance	This study
22	c.2600T>A	p.L867H	IQ motif	T	B	D	L	0.407	23.5	−	0.000039	0	Uncertain_Significance	This study
22	c.2651T>C	p.M884T	−	T	B	D	M	0.662	16.9	-	0	0	Uncertain_Significance	This study
22	c.2665G>A	p.A889T	−	T	B	D	M	0.475	23.4	-	0	0	Uncertain_Significance	This study
23	c.2708A>C	p.Q903P	−	T	B	D	L	0.682	24.1	-	0	0	Uncertain_Significance	This study
23	c.2717G>C	p.R906P	−	D	P	D	M	0.623	25.5	-	0	0	Uncertain_Significance	This study
23	c.2837_2839del	p.M946_F947delinsL	−	−	−	−	−	−	−	−	0	0	Uncertain_Significance	This study
23	c.2839T>G	p.F947V	−	D	D	D	M	0.845	28.2	−	0	0	Uncertain_Significance	This study
31	c.4118G>A	p.R1373Q	FERM domain	D	D	D	M	0.638	34.0	−	0.000040	0	Uncertain_Significance	This study
32	c.4157A>G	p.D1386G	FERM domain	D	B	D	M	0.433	25.2	−	0	0.000004	Uncertain_Significance	This study
37	c.5138C>T	p.T1713M	SH3 domain	D	D	D	M	0.747	34.0	−	0.000013	0.000019	Uncertain_Significance	This study
48	c.6529G>A	p.G2177R	FERM domain	D	D	D	M	0.751	34.0	−	0	0	Uncertain_Significance	This study

All variants are indicated on NM_000260.

AA, amino acid; PP2, PolyPhen2; MutTaster, mutation taster, MutAssessor, mutation assessor; D, deleterious (SIFT); T, tolerant (SIFT); D, probably damaging (PP2); P, possibly damaging (PP2); B, benign (PP2); D, disease-causing (MutTaster); N, polymorphism (MutTaster); H, high (MutAssessor); M, medium (MutAssessor); L, low (MutAssessor).

**Table 2:** *MYO7A* candidate variants for DFNB2 and USH1B identified in this study.

Exon	Base change	AA change	Domain	SIFT	PP2HV	MutTaster	MutAssessor	REVEL	CADD Phred	dbscSNV	TOMMO 38KJPN	gnomAD exome	Pathogenicity	References
3	c.52C>T	p.Q18*	−	−	−	A	−	−	36	−	0	0	AR_Pathogenic	Cremers FP et al., J Med Genet. 2007;44:153–60.(USH1B)
5	c.322delT	p.Y108Tfs*38	−	−	−	−	−		−	−	0	0	AR_Pathogenic	This study
5	c.448C>T	p.R150*	−	−	−	A	−	−	37	−	0.000013	0	AR_Pathogenic	Weil D et al., Nature. 1995;374:60–1. (USH1B)
6	c.473G>A	p.G158E	Myosin head,	D	D	D	H	0.971	26.1	−	0	0	AR_Likely_Pathogenic	This study
7	c.635G>A	p.R212H	Myosin head	D	D	A	H	0.898	34	−	0.000026	0.000012	AR_Pathogenic	Weil D et al., Nature. 1995;374:60–1. (USH1B)
8	c.785_788del	p.S263Rfs*24	−	−	−	−	−	−	−	−	0.000013	0	AR_Likely_Pathogenic	This study
8	c.849+1G>A	Splicing	−	−	−	D	−	−	26	1.0000	0	0.0000044	AR_Likely_Pathogenic	This study
9	c.940G>A	p.E314K	Myosin head	D	D	D	M	0.825	34	−	0.000026	0.000036	Uncertain_Significance	This study
12	c.1343+1G>A	Splicing	−	−	−	D	−	−	27.1	1.0000	0	0	AR_Pathogenic	Rong W et al., PLoS One. 2014;9:e97808. (USH1B)
13	c.1369G>A	p.A457T	Myosin head	D	P	D	L	0.636	25.8	−	0.000168	0.000008	AR_Likely_Pathogenic	Uehara N et al., J Hum Genet. 2022;67:223–230
13	c.1431T>A	p.Y477*	−	−	−	A	−	−	35	−	0	0	AR_Likely_Pathogenic	Kim Y S et al., Sci Rep. 2022;12:e12335
13	c.1537G>C	p.E513Q	Myosin head	D	D	D	M	0.713	27.9	−	0.000052	0	Uncertain_Significance	This study
14	c.1667G>T	p.G556V	Myosin head	D	D	D	H	0.844	25.6	−	0.000013	0	AR_Likely_Pathogenic	Bakondi B et al. Mol Ther. 2016;24:556–63. (USH1B)
14	c.1617dupC	p.K542Qfs*5	−	−	−	−	−	−	−	−	0.000065	0.000008	AR_Pathogenic	Bharadwaj AK et al., Exp Eye Res. 2000;71:173–81. (USH1B)
15	c.1708C>T	p.R570*	−	−	−	A	−	−	42	−	0	0	AR_Pathogenic	Yoshimura H et al., PLoS One. 2014;9:e90688. (USH1B)
16	c.1820C>T	p.S607L	Myosin head	T	P	D	N	0.425	26.6	−	0.000245	0.000018	Uncertain_Significance	This study
17	c.2023C>T	p.R675C	Myosin head	D	D	D	H	0.893	35	−	0.000284	0.000020	Uncertain_Significance	Miyagawa M et al., PLoS One. 2013;8:e71381. (DFNB2)
17	c.2005C>T	p.R669*	−	−	−	A	−	−	39	−	0	0.000016	AR_Pathogenic	Liu XZ et al., Am J Hum Genet. 1998;63:909–12. (USH1B)
17	c.2074G>A	p.V692M	Myosin head	T	P	D	L	0.778	24.5	−	0	0	AR_Pathogenic	Yoshimura H et al., PLoS One. 2014;9:e90688. (USH1B)
17	c.2079_2080insCC	p.A695Rfs*28	−	−	−	−	−	−	−	−	0	0	AR_Pathogenic	This study
18	c.2115C>A	p.C705*	−	−	−	A	−	−	37	−	0.000193	0.000012	AR_Pathogenic	Yoshimura H et al., PLoS One. 2014;9:e90688. (USH1B)
21	c.2471_2472insCCGCGCCTATCTGGTGCGCAAGGCCTTCCGCCA	p.R836_L837insAYLVRKAFRHR	−	−	−	−	−	−	−	−	0	0	Uncertain_Significance	This study
23	c.2777dupT	p.E927Gfs*9	−	−	−	−	−	−	−	−	0	0	AR_Likely_Pathogenic	This study
27	c.3475G>A	p.G1159S	MyTH4 domain	D	D	D	M	0.929	34	−	0.000542	0.000089	AR_Pathogenic	This study
28	c.3508G>A	p.E1170K	MyTH4 domain	D	D	D	H	0.964	32	−	0	0.000020	AR_Pathogenic	Cuevas JM et al., Hum Mutat. 1999;14:181. (USH1B)
29	c.3718C>T	p.R1240W	MyTH4 domain	D	D	D	H	0.92	32	−	0.000013	0	AR_Pathogenic	Cremers FP et al., J Med Genet. 2007;44:153–60. (USH1B)
31	c.3932delC	p.L1312Wfs*87	−	−	−	−	−		−	−	0	0	AR_Likely_Pathogenic	This study
31	c.4039C>G	p.R1347G	FERM domain	D	D	D	M	0.93	31	−	0.000193	0	Uncertain_Significance	This study
34	c.4482_4483insTG	p.W1495Cfs*55	−	−	−	−	−		−	−	0	0	AR_Pathogenic	Yoshimura H et al., PLoS One. 2014;9:e90688. (USH1B)
34	c.4490G>T	p.G1497V	−	D	D	D	H	0.962	28.4	−	0	0	Uncertain_Significance	This study
34	c.4510C>T	p.Q1504*	−	−	−	A	−	−	47	−	0	0	AR_Pathogenic	Yoshimura H et al., J Hum Genet. 2016;61:419–22. (USH1B)
36	c.4960G>T	p.E1654*	−	−	−	A	−	−	52	−	0	0	AR_Likely_Pathogenic	This study
37	c.5106delG	p.A1703Rfs*28	−	−	−	−	−	−	−	−	0	0	AR_Likely_Pathogenic	This study
38	c.5320T>C	p.F1774L	MyTH4 domain	D	B	D	M	0.935	24.3	−	0	0	AR_Likely_Pathogenic	Yoshimura H et al., Int J Pediatr Otorhinolaryngol. 2013;77:298–302.(USH1B)
40	c.5481-1G>C	Splicing	−	−	−	D	−	−	24.2	1.0000	0.000103	0	AR_Likely_Pathogenic	Suga A et al., Hum Mutat. 2022;12:2251–2264. (USH1B)
40	c.5617C>T	p.R1873W	MyTH4 domain	D	D	D	M		34	−	0.000116	0.000012	AR_Pathogenic	Roux AF et al., J Med Genet. 2006;43:763–8. (USH1B)
40	c.5636+1G>T	Splicing	−	−	−	D	−	−	25	1.0000	0	0	AR_Likely_Pathogenic	Yoshimura H et al., J Hum Genet. 2016;61:419–22. (USH1B)
41	c.5660C>T	p.P1887L	MyTH4 domain	D	D	D	M	0.918	29.5	−	0.000013	0.000012	AR_Pathogenic	Bharadwaj AK et al., Exp Eye Res. 2000;71:173–81. (USH1B)
43	c.5866_5867insTTCCTGAGAATGACTTCTTCTTTGAC	p.V1966Lfs*13	−	−	−	−	−	−	−	−	0	0	AR_Pathogenic	This study
43	c.5899C>T	p.R1967*	−	−	−	A	−	−	46	−	0.0000129	0.000012	AR_Pathogenic	Bujakowska KM et al., Invest Ophthalmol Vis Sci. 2014;55:8488–96. (USH1B)
43	c.5930G>A	p.R1977Q	FERM domain	D	P	D	M	0.639	25.4	−	0.000387	0.000069	Uncertain_Significance	Kim Y J et al., Genes (Basel). 2021;12(5):675. (USH1B)
45	c.6204_6205del	p.I2069Pfs*6	−	−	−	−	−	−	−	−	0	0	AR_Pathogenic	Yoshimura H et al., PLoS One. 2014;9:e90688. (USH1B)
46	c.6321G>A	p.W2107*	−	−	−	A	−	−	47	−	0.000039	0	AR_Pathogenic	Yoshimura H et al., PLoS One. 2014;9:e90688. (USH1B)
48	c.6478T>G	p.W2160G	FERM domain	D	D	D	M	0.911	29.7	−	0.000077	0	AR_Likely_Pathogenic	Mutai H et al., Orphanet J Rare Dis. 2013;8:172. (DFNB2)
48	c.6542T>C	p.L2181P	FERM domain	D	D	D	M	0.939	28.3	−	0	0	Uncertain_Significance	Yoshimura H et al., J Hum Genet. 2016;61:419–22. (USH1B)
48	c.6551C>T	p.T2184M	FERM domain	D	D	D	M	0.823	34	−	0	0	Uncertain_Significance	Carss K J et al., Am J Hum Genet. 2017;100:75–90. (USH1B)
48	c.6558+1G>C	Splicing	−	−	−	D	−	–	25.5	1.0000	0	0	AR_Likely_Pathogenic	This study

All variants are indicated on NM_000260.

AA, amino acid; PP2, PolyPhen2; MutTaster, mutation taster; MutAssessor, mutation assessor; D, deleterious (SIFT); T, tolerant (SIFT); D, probably damaging (PP2); P, Possibly Damaging (PP2); B, Benign (PP2); D, Disease-causing (MutTaster); A, disease-causing automatic (MutTaster); H, high (MutAssessor); M, medium (MutAssessor); L, low (MutAssessor); N, neutral (MutAssessor).

The prevalence of *MYO7A*-associated HL in this large Japanese HL cohort was 1.36% (137/10,047). The prevalence of *MYO7A*-assocciated ADNSHL in autosomal dominant or maternal inheritance HL patients was 4.06% (91/2243). Similarly, the prevalence of *MYO7A*-associated ARNSHL patients in autosomal recessive or sporadic HL patients was 0.38% (25/6163), and 0.32% (21/6163) for cases of *MYO7A*-associated Usher syndrome.

### Clinical features of DFNA11, DFNB2 and USH1B patients

The detailed clinical features of DFNA11, DFNB2 and USH1B patients are shown in Tables 3, 4 and 5, respectively. Family segregation analysis results were shown in Supplemental Figure 1, 2 and 3.

**Table 3:** Clinical characteristics of autosomal dominant *MYO7A*-assocciated HL patients (DFNA11).

Family no.	Relationship	Base change	AA change	Hereditary	Onset	Age	Sex	Tinnitus	Vertigo	Progression	Severity	Configuration	HA	CI	Onset of visual symptom	Diagnosis of RP	Neck supported when sitting (month)	Walking started (month)	Caloric testing
1		c.[420C>G;5503G>C]	p.[N140K;E1835Q]	AD	60	62	F	+	+	+	N/A	N/A	+	−	−	−	N/A	N/A	−
2		c.[420C>G;5503G>C]	p.[N140K;E1835Q]	AD	38	43	M	+	−	−	Mild	Flat	−	−	−	−	N/A	N/A	−
3		c.[420C>G;5503G>C]	p.[N140K;E1835Q]	AD	32	41	M	−	−	+	Mild	Ascending	−	−	−	−	N/A	N/A	−
4		c.[439C>T;1436T>C]	p.[R147C;L479P]	AD	30	53	M	+	+	+	Moderate	Ascending	+	−	−	−	N/A	N/A	−
5		c.[479C>G;2947G>T]	p.[S160C;D983Y]	AD	8	N/A	F	+	+	+	N/A	N/A	−	Bi	−	−	N/A	N/A	Rt.CP
6		c.[479C>G;2947G>T]	p.[S160C;D983Y]	AD	9	37	F	+	−	+	Moderate	Gently sloping	−	−	−	−	N/A	N/A	−
7	Proband	c.[479C>G;2947G>T]	p.[S160C;D983Y]	AD	7	10	F	−	−	+	Mild	Steeply sloping	−	−	−	−	Not delayed	Not delayed	−
	Father	c.[479C>G;2947G>T]	p.[S160C;D983Y]	AD	13	44	M	+	−	+	Moderate	U-shaped	+	−	−	−	Not delayed	Not delayed	−
8	Proband	c.[479C>G;2947G>T]	p.[S160C;D983Y]	AD	10	33	M	−	−	+	Moderate	Gently sloping	+	−	−	−	6	15	−
	Father	c.[479C>G;2947G>T]	p.[S160C;D983Y]	AD	30	68	M	−	−	+	Profound	Flat	+	Rt	−	−	N/A	N/A	−
9		c.[479C>G;2947G>T]	p.[S160C;D983Y]	AD	13	43	M	+	−	+	Moderate	U-shaped	+	−	−	−	3	13	−
10		c.[479C>G;2947G>T]	p.[S160C;D983Y]	AD	6	36	F	+	−	+	Moderate	Gently sloping	+	−	−	−	N/A	N/A	−
11		c.[479C>G;2947G>T]	p.[S160C;D983Y]	AD	N/A	38	M	−	−	+	Moderate	Steeply sloping	N/A	N/A	−	−	N/A	N/A	−
12		c.[479C>G;2947G>T]	p.[S160C;D983Y]	Spo	10	31	F	+	+	+	Severe	Steeply sloping	−	−	−	−	4	12	−
13		c.[479C>G;2947G>T]	p.[S160C;D983Y]	AD	10	67	M	+	−	+	Moderate	Ascending	+	−	−	−	N/A	N/A	−
14		c.[479C>G;2947G>T]	p.[S160C;D983Y]	AD	16	25	F	+	−	+	Moderate	Flat	+	−	−	−	N/A	N/A	−
15	Proband	c.547T>C	p.S183P	AD	45	71	M	+	−	+	Severe	Steeply sloping	+	Lt	−	−	N/A	N/A	−
	Sister	c.547T>C	p.S183P	AD	N/A	79	F	−	−	+	N/A	N/A	N/A	N/A	−	−	N/A	N/A	−
16		c.689C>T	p.A230V	Spo (de novo)	11	21	F	+	−	+	Mild	Ascending	+	−	−	−	N/A	N/A	−
17	Proband	c.1436T>C	p.L479P	AD	13	50	F	+	−	+	Moderate	Ascending	+	−	−	−	N/A	N/A	−
	Daughter	c.1436T>C	p.L479P	AD	13	21	F	−	−	−	Mild	Ascending	−	−	−	−	N/A	N/A	−
18		c.1436T>C	p.L479P	Spo	31	42	M	−	−	+	Moderate	Flat	N/A	N/A	−	−	N/A	N/A	−
19	Proband	c.1436T>C	p.L479P	AD	28	44	M	−	−	+	Mild	Ascending	+	−	−	−	N/A	N/A	−
	Daughter	c.1436T>C	p.L479P	AD	3	5	F	N/A	−	N/A	Normal	Ascending	−	−	−	−	N/A	N/A	−
20	Proband	c.1436T>C	p.L479P	AD	10	37	M	+	−	+	Moderate	Ascending	N/A	N/A	−	−	N/A	N/A	−
	Father	c.1436T>C	p.L479P	AD	N/A	71	M	N/A	N/A	N/A	Severe	Flat	N/A	N/A	−	−	N/A	N/A	−
21	Proband	c.1436T>C	p.L479P	AD	46	54	F	+	−	+	N/A	N/A	+	−	−	−	N/A	N/A	−
	Brother	c.1436T>C	p.L479P	AD	0	45	M	−	−	+	Moderate	Ascending	+	−	−	−	N/A	N/A	−
	Nephew	c.1436T>C	p.L479P	AD	0	20	F	−	−	−	Normal	Ascending	−	−	−	−	N/A	N/A	−
	Daughter	c.1436T>C	p.L479P	AD	N/A	29	F	−	+	−	Normal	Ascending	−	−	−	−	N/A	N/A	normal
	Son	c.1436T>C	p.L479P	AD	N/A	27	M	−	−	−	Normal	Ascending	−	−	−	−	N/A	N/A	−
22		c.1436T>C	p.L479P	AD	20	52	M	+	−	+	Severe	U-shaped	+	−	−	−	4	12	−
23		c.1436T>C	p.L479P	AD	15	38	F	−	−	+	Moderate	flat	+	N/A	−	−	N/A	N/A	−
24		c.1436T>C	p.L479P	AD	47	55	M	+	+	+	Moderate	Ascending	+	−	−	−	N/A	N/A	−
25		c.1436T>C	p.L479P	AD	55	57	F	−	−	+	Mild	Ascending	+	−	−	−	N/A	N/A	−
26		c.1436T>C	p.L479P	AD	N/A	32	F	−	−	+	Mild	Ascending	−	−	−	−	N/A	N/A	−
27		c.1436T>C	p.L479P	AD	55	75	F	+	−	+	Profound	Flat	+	Lt	−	−	N/A	N/A	−
28	Proband	c.1436T>C	p.L479P	AD	6	10	M	+	+	N/A	Mild	Ascending	N/A	N/A	−	−	N/A	N/A	−
	Mother	c.1436T>C	p.L479P	AD	25	N/A	F	+	−	N/A	Mild	Ascending	N/A	N/A	−	−	N/A	N/A	−
29		c.1966C>G	p.L656V	AD	42	47	M	+	−	+	Moderate	Gently sloping	N/A	N/A	−	−	N/A	N/A	−
30		c.1966C>G	p.L656V	Unknown	54	64	F	+	−	N/A	Severe	Flat	+	−	−	−	N/A	N/A	−
31		c.1966C>G	p.L656V	AD	40	56	M	+	+	N/A	Moderate	Flat	+	−	−	−	N/A	N/A	−
32		c.1966C>G	p.L656V	AD	20	66	F	+	−	+	Moderate	Flat	+	−	−	−	N/A	N/A	−
33		c.1966C>G	p.L656V	Spo	10	41	M	−	−	+	Severe	Ascending	+	−	−	−	N/A	N/A	−
34	Proband	c.1978G>A	p.G660R	AD	6	7	F	−	−	−	Mild	Ascending	+	−	−	−	N/A	N/A	−
	Mother	c.1978G>A	p.G660R	AD	28	N/A	F	N/A	N/A	N/A	N/A	N/A	+	−	−	−	N/A	N/A	−
35	Proband	c.1978G>A	p.G660R	AD	7	8	F	−	−	N/A	Mild	Gently sloping	−	−	−	−	N/A	N/A	−
	Father	c.1978G>A	p.G660R	AD	5	42	M	+	−	+	Moderate	Ascending	+	−	−	−	N/A	N/A	−
36		c.2003G>A	p.R668H	Spo	N/A	63	M	−	−	−	Moderate	U-shaped	+	−	−	−	N/A	N/A	−
37		c.2003G>A	p.R668H	AD	25	41	F	+	+	+	Mild	Ascending	−	−	−	−	N/A	N/A	Bi.CP
38	Proband	c.2185A>G	p.K729E	AD	30	69	M	−	−	+	Severe	Steeply sloping	+	−	−	−	N/A	N/A	−
	Son	c.2185A>G	p.K729E	AD	10	42	M	−	+	+	Severe	Steeply sloping	+	−	−	−	N/A	N/A	−
	Son	c.2185A>G	p.K729E	AD	30	42	M	−	−	+	Moderate	Steeply sloping	−	−	−	−	N/A	N/A	−
	Grand daughter	c.2185A>G	p.K729E	AD	10	13	F	−	−	+	Mild	Gently sloping	−	−	−	−	N/A	N/A	−
39	Proband	c.2558G>A	p.R853H	AD	25	36	F	+	−	+	Moderate	Flat	−	−	−	−	N/A	N/A	−
	Daughter	c.2558G>A	p.R853H	AD	6	15	F	+	−	−	Mild	Flat	−	−	−	−	N/A	N/A	−
40	Proband	c.2558G>A	p.R853H	AD	N/A	40	M	−	−	N/A	Moderate	Gently sloping	−	−	−	−	N/A	N/A	−
	Daughter	c.2558G>A	p.R853H	AD	6	7	F	−	−	N/A	Mild	Gently sloping	−	−	−	−	N/A	N/A	−
	Daughter	c.2558G>A	p.R853H	AD	15	15	F	−	−	N/A	Mild	Gently sloping	−	−	−	−	N/A	N/A	−
	Father	c.2558G>A	p.R853H	AD	N/A	71	M	N/A	N/A	N/A	Profound	Flat	+	N/A	−	−	N/A	N/A	−
41	Proband	c.2558G>A	p.R853H	AD	N/A	8	M	−	−	+	Mild	Steeply sloping	−	−	−	−	N/A	N/A	−
	Father	c.2558G>A	p.R853H	AD	N/A	36	M	−	−	+	Mild	Gently sloping	−	−	−	−	N/A	N/A	−
	Sister	c.2558G>A	p.R853H	AD	N/A	11	F	−	−	+	Mild	Gently sloping	−	−	−	−	N/A	N/A	−
42	Proband	c.2558G>A	p.R853H	AD	56	60	F	+	−	+	Mild	Flat	N/A	N/A	−	−	N/A	N/A	−
	Daughter	c.2558G>A	p.R853H	AD	26	28	F	+	+	+	Mild	Ascending	N/A	N/A	−	−	N/A	N/A	−
43		c.2558G>A	p.R853H	AD	25	65	F	+	−	+	Profound	Gently sloping	−	Bi	−	−	N/A	N/A	−
44	Proband	c.2558G>A	p.R853H	AD	10	18	F	−	−	+	Mild	Gently sloping	N/A	−	−	−	N/A	N/A	−
	Father	c.2558G>A	p.R853H	AD	N/A	55	M	−	−	+	Moderate	Gently sloping	N/A	−	−	−	N/A	N/A	−
45	Proband	c.2558G>T	p.R853L	AD	0	0	M	N/A	N/A	N/A	Moderate	Gently sloping	N/A	N/A	−	−	N/A	N/A	−
	Father	c.2558G>T	p.R853L	AD	N/A	33	M	−	−	+	Moderate	Steeply sloping	N/A	N/A	−	−	N/A	N/A	−
46		c.2600 T>A	p.L867H	AD	55	70	F	−	−	+	Moderate	Flat	+	−	−	−	N/A	N/A	−
47	Proband	c.2651 T>C	p.M884T	AD	10	33	M	+	−	+	Moderate	Gently sloping	+	−	−	−	N/A	N/A	−
	Mother	c.2651 T>C	p.M884T	AD	3	60	F	+	−	+	Moderate	Gently sloping	+	−	−	−	N/A	N/A	−
48	Proband	c.2665G>A	p.A889T	AD	15	34	F	+	−	+	Mild	Gently sloping	+	−	−	−	N/A	N/A	−
	Mother	c.2665G>A	p.A889T	AD	50	66	F	−	−	+	Moderate	Flat	+	−	−	−	N/A	N/A	−
49		c.2665G>A	p.A889T	AD	30	42	M	+	−	+	Severe	U-shaped	N/A	N/A	−	−	N/A	N/A	−
50		c.2665G>A	p.A889T	AD	6	9	M	−	−	+	Moderate	Gently sloping	+	−	−	−	N/A	N/A	−
51		c.2708A>C	p.Q903P	AD	15	48	F	+	−	+	Moderate	Steeply sloping	+	−	−	−	N/A	N/A	−
52		c.2708A>C	p.Q903P	AD	25	50	M	+	+	+	Moderate	Flat	+	−	−	−	N/A	N/A	−
53		c.2717G>C	p.R906P	AD	N/A	33	F	+	+	+	Severe	Flat	+	−	−	−	not delayed	not delayed	−
54		c.2837_2839del	p.M946_F947delinsI	AD	6	32	F	+	−	+	Severe	Gently sloping	+	−	−	−	N/A	N/A	−
55	Proband	c.2839T>G	p.F947V	AD	N/A	64	F	+	−	+	Profound	Flat	+	+	−	−	N/A	N/A	−
	Son	c.2839T>G	p.F947V	AD	N/A	36	M	N/A	N/A	N/A	Mild	Gently sloping	N/A	N/A	−	−	N/A	N/A	−
56	Proband	c.2839T>G	p.F947V	AD	45	50	M	−	−	+	Moderate	Steeply sloping	−	−	−	−	N/A	N/A	−
	Daughter	c.2839T>G	p.F947V	AD	15–18	24	F	N/A	N/A	N/A	Mild	Flat	+	−	−	−	N/A	N/A	−
	Son	c.2839T>G	p.F947V	AD	N/A	23	M	N/A	N/A	N/A	Mild	Flat	−	−	−	−	N/A	N/A	−
57		c.4118G>A	p.R1373Q	AD	45	68	M	+	−	+	Moderate	Steeply sloping	+	−	−	−	N/A	N/A	−
58	Proband	c.4157A>G	p.D1386G	AD	0	3	M	−	−	+	Mild	Steeply sloping	+	−	−	−	N/A	N/A	−
	Father	c.4157A>G	p.D1386G	AD	11	35	M	+	−	+	N/A	N/A	N/A	N/A	−	−	N/A	N/A	−
59		c.5138C>T	p.T1713M	AD	30	39	M	+	−	+	Moderate	Steeply sloping	+	−	−	−	N/A	N/A	−
60		c.6529G>A	p.G2177R	AD	6	34	F	−	−	−	N/A	N/A	+	−	−	−	Not delayed	Not delayed	−

All variants are indicated on NM_000260.

HA, hearing aid; CI, cochlear implant; AD, autosomal dominant; Spo, sporadic; M, male; F, female; N/A, data not available; Rt., right; Lt., left; Bi., bilateral; CP, canal paresis.

**Table 4:** Clinical characteristics of autosomal recessive *MYO7A*-assocciated HL patients (DFNB2).

Family no.	Relationship	Base change		AA change		Hereditary	Onset of HL	Age at Genetic testing	Sex	Tinnitus	Vertigo	Progression	Severity	Configuration	HA	CI	Onset of visual symptom	Diagnosis of RP	Neck supported when sitting (month)	Walking started (month)	Caloric testing
1		c.473G>A	c.2777dupT	p.G158E	p.E927Gfs*9	Spo	0	2	M	-	−	−	Profound	Flat	+	Rt	−	−	4	19	N/A
2	Proband	c.635G>A	c.3475G>A	p.R212H	p.G1159S	Spo	0	11	F	−	−	−	Severe	U-shaped	N/A	N/A	N/A	N/A	N/A	N/A	N/A
	Brother	c.635G>A	c.3475G>A	p.R212H	p.G1159S	Spo	0	13	M	+	−	+	Moderate	Ascending	N/A	N/A	N/A	N/A	N/A	N/A	N/A
3	Proband	c.849+1G>A	c.3475G>A	Splicing	p.G1159S	AR	0	5	M	−	−	−	Moderate	Ascending	+	−	N/A	N/A	4	13	N/A
	Brother	c.849+1G>A	c.3475G>A	Splicing	p.G1159S	AR	0	9	M	−	−	−	Mild	Flat	+	−	N/A	N/A	3	14	N/A
4		c.940G>A	c.3932delC	p.E314K	p.L1312Wfs*87	AR	3	25	F	−	−	+	Severe	Gently sloping	+	planed	N/A	N/A	N/A	N/A	N/A
5		c.1369G>A	c.1369G>A	p.A457T	p.A457T	Spo	0	23	F	−	−	+	Moderate	Ascending	N/A	N/A	N/A	N/A	N/A	N/A	N/A
6		c.1369G>A	c.1369G>A	p.A457T	p.A457T	Spo	20	41	F	+	+	+	Severe	Flat	−	Bi	−	−	4	12	N/A
7		c.1369G>A	c.1667G>T	p.A457T	p.G556V	Spo	7	46	M	+	−	+	Severe	Flat	−	Bi	−	49	Not delayed	Not delayed	Bi.CP sus
8		c.1431T>A	c.3475G>A	p.Y477X	p.G1159S	Unknown	0	3	F	−	−	+	Moderate	Flat	+	−	−	−	3	12	N/A
9		c.1537G>C	c.1820C>T	p.E513Q	p.S607L	Spo	42	43	F	−	−	−	Mild	Steeply sloping	−	−	−	−	N/A	N/A	N/A
10		c.1537G>C	c.1820C>T	p.E513Q	p.S607L	Spo	40	46	M	+	+	+	Moderate	Steeply sloping	−	−	35	−	N/A	N/A	N/A
11		c.1537G>C	c.5481-1G>C	p.E513Q	Splicing	Spo	0	1	F	−	−	−	Moderate	Steeply sloping	+	−	N/A	N/A	Not delayed	14	N/A
12		c.1537G>C	c.5930G>A	p.E513Q	p.R1977Q	AD	20	56	F	−	+	−	Severe	Gently sloping	+	Rt	−	−	N/A	N/A	Normal
13		c.1537G>C	c.6321G>A	p.E513Q	p.W2107X	Spo	10	24	F	+	−	+	Moderate	Steeply sloping	+	−	−	−	N/A	N/A	N/A
14		c.2023C>T	c.2471_2472insCCGCGCCTATCTGGTGCGCAAGGCCTTCCGCCA	p.R675C	p.R836_L837insAYLVRKAFRHR		9	40	F	+	+	+	Severe	Ascending	+	Rt	N/A	N/A	N/A	N/A	Bi.CP
15	Proband	c.2115C>A	c.3475G>A	p.C705X	p.G1159S	AR	0	1	M	−	−	+	Moderate	Ascending	+	−	−	−	2	14	N/A
	Brother	c.2115C>A	c.3475G>A	p.C705X	p.G1159S	AR	0	2	M	−	−	+	Moderate	Flat	+	planed	−	−	3	14	N/A
16		c.3475G>A	c.3475G>A	p.G1159S	p.G1159S	Spo	3	25	F	−	−	+	Severe	Gently sloping	+	Lt	22	23	N/A	N/A	Bi.CP
17		c.3475G>A	c.3475G>A	p.G1159S	p.G1159S	AD	10	38	M	−	−	+	Moderate	Flat	+	N/A	N/A	N/A	N/A	N/A	N/A
18	Proband	c.3475G>A	c.5617C>T	p.G1159S	p.R1873W	AR	0	0	M	−	−	−	Mild	Gently sloping	+	−	−	−	Not delayed	Not delayed	N/A
	Brother	c.3475G>A	c.5617C>T	p.G1159S	p.R1873W	AR	0	1	M	−	−	+	Moderate	Flat	+	−	−	−	Not delayed	Not delayed	N/A
	Brother	c.3475G>A	c.5617C>T	p.G1159S	p.R1873W	AR	0	2	M	−	−	+	Moderate	U-shaped	+	N/A	N/A	N/A	N/A	N/A	N/A
19		c.3475G>A	c.6321G>A	p.G1159S	p.W2107X	Spo	0	3	M	−	−	−	Moderate	Flat	+	N/A	N/A	N/A	N/A	N/A	N/A
20		c.4039C>G	c.4039C>G	p.R1347G	p.R1347G	Spo	3	38	M	+	−	+	Profound	U-shaped	+	−	−	−	N/A	11	N/A

All variants are indicated on NM_000260.

HA, hearing aid, CI, cochlear implant; AD, autosomal dominant; Spo, sporadic; M, male; F, female; N/A, data not available; Rt., right; Lt., left; Bi., bilateral; CP, canal paresis.

**Table 5:** Clinical characteristics of *MYO7A*-assocciated Usher syndrome patients (USH1B).

Family no.	Relationship	Base change		AA Change		Hereditary	Onset of HL	Age at genetic testing	Sex	Tinnitus	Vertigo	Progression	Severity	Configuration	HA	CI	Onset of visual symptom	Diagnosis of RP	Neck supported when sitting (month)	Walking started (month)	Caloric testing
1		c.52C>T	c.2115C>A	p.Q18X	p.C705X	Spo	0	0	M	−	−	−	Severe	Flat	+	Rt	5	3	3	17	N/A
2		c.52C>T	c.5320T>C	p.Q18X	p.F1774L	Spo	0	7	M	−	−	+	Profound	Flat	−	Bi	7	7	3	12	N/A
3		c.332delT	c.4482_4483insTG	p.Y108Tfs*38	p.W1495Cfs*55	Spo	0	22	F	−	−	−	Profound	Flat	−	Rt	15	6	N/A	N/A	N/A
4		c.448C>T	c.6321G>A	p.R150X	p.W2107X	Spo	0	0	M	−	−	−	Profound	Flat	−	Bi	−	−	7	21	N/A
5	Proband	c.785_788del	c.4960G>T	p.S263Rfs*24	p.E1654X	AR	0	48	M	−	−	−	Profound	Flat	+	−	5	15	N/A	N/A	N/A
	Sister	c.785_788del	c.4960G>T	p.S263Rfs*24	p.E1654X	AR	3	54	F	−	+	−	Profound	Flat	+	−	13	13	N/A	N/A	N/A
6		c.1343+1G>A	c.5866_5867insTTCCTGAGAATGACTTCTTCTTTGAC	Splicing	p.V1966Lfs*13	Spo	0	2	M	−	−	+	Severe	Flat	−	Bi	−	−	4	24	N/A
7		c.1617dupC	c.6478T>G	p.K542Qfs*5	p.W2160G	Spo	0	13	M	−	+	−	Profound	Flat	−	Bi	10	10	N/A	18	Bi.CP
8		c.1708C>T	c.1708C>T	p.R570X	p.R570X	Unknown	0	60	F	N/A	−	−	N/A	N/A	+	N/A	before 7	7	N/A	N/A	N/A
9	Proband	c.2005C>T	c.5660C>T	p.R669X	p.P1887L	AR	0	4	M	−	−	−	Profound	Flat	+	Bi	−	−	N/A	N/A	N/A
	Sister	c.2005C>T	c.5660C>T	p.R669X	p.P1887L	AR	0	1	F	−	−	−	Profound	Gently sloping	+	Bi	−	−	N/A	N/A	N/A
10		c.2074G>A	c.2074G>A	p.V692M	p.V692M	AR	0	60	M	−	−	−	Profound	Flat	−	−	6	15	N/A	N/A	N/A
11		c.2079_2080insCC	c.5899C>T	p.A695Rfs*28	p.R1967X	Spo	0	2	M	−	−	−	N/A	N/A	−	−	−	−	N/A	N/A	N/A
12		c.2115C>A	c.3718C>T	p.C705X	p.R1240W	Spo	2	16	F	−	−	−	Profound	Flat	+	Lt	6	11	4	28	N/A
13		c.2115C>A	c.6542 T>C	p.C705X	p.L2181P	Unknown	0	17	M	−	−	N/A	Profound	Flat	+	Bi	Before 12	12	N/A	N/A	Bi.CP
14	Proband	c.3508G>A	c.6551C>T	p.R1170K	p.T2184M	AR	0	14	M	−	−	−	Severe	Flat	+	Bi	N/A	14	N/A	N/A	Bi.CP
	Brother	c.3508G>A	c.6551C>T	p.R1170K	p.T2184M	AR	0	11	M	−	−	−	Severe	Gently sloping	+	Bi	7	7	N/A	N/A	Bi.CP
15		c.4490G>T	c.5106delG	p.G1497V	p.A1703Rfs*28	Spo	0	7	M	−	−	−	Profound	Flat	+	Bi	5	7	5	20	N/A
16		c.4510C>T	c.5636+1G>T	p.Q1504X	Splicing	Spo	0	2	M	−	−	−	Profound	Flat	+	−	N/A	N/A	8	24	N/A
17		c.6204_6205del	c.6204_6205del	p.I2069Pfs*6	p.I2069Pfs*6	AR	0	47	F	−	+	−	Profound	Flat	+	−	+	N/A	Delayed	Delayed	Bi.CP
18		c.6558+1G>C	c.6558+1G>C	Splicing	Splicing	Spo	0	41	F	−	−	−	Profound	Flat	+	−	5	N/A	N/A	N/A	N/A

All variants are indicated on NM_000260.

HA, hearing aid; CI, cochlear implant; AD, autosomal dominant; Spo, sporadic; M, male; F, female; N/A, data not available; Rt., right; Lt., left; Bi., bilateral; CP, canal paresis.

**This table also includes the patients under 5 years old who carried previously reported biallelic USH1B variants without visual symptoms if they showed delays in sitting and/or walking.

**Figure 1:**
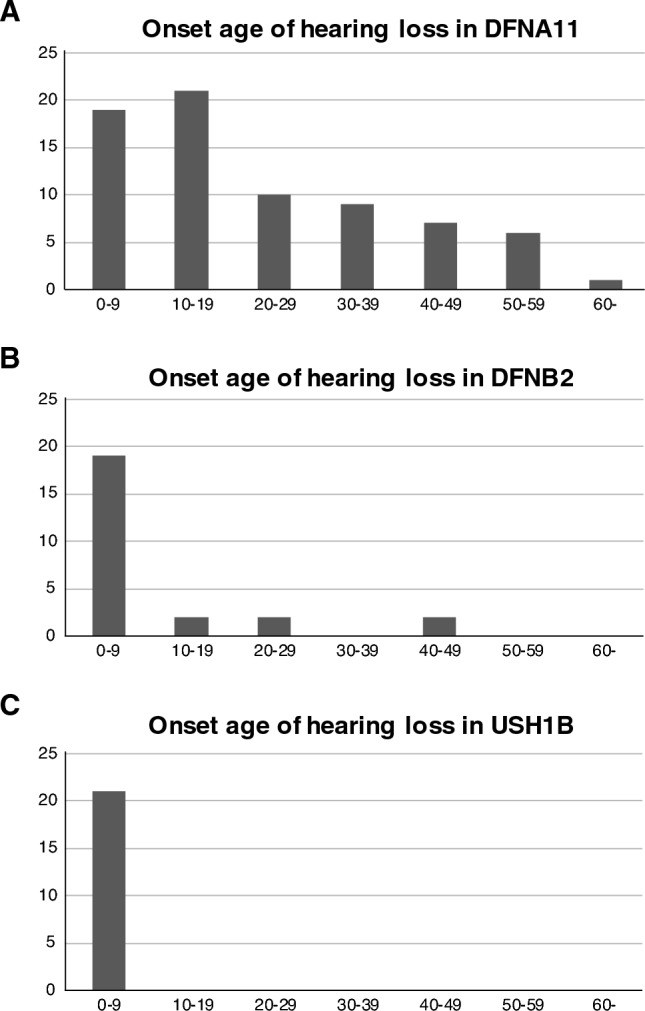
Onset age of hearing loss in patients with (**A**) DFNA11 variants, (**B**) DFNB2 variants, and (**C**) USH1B variants. There was a tendency for onset age to differ depending on variant type. About half of the DFNA11 patients developed or became aware their HL in their first or second decade, whereas HL onset in about half was after twenty years of age. Almost all DFNB2 cases showed congenital or early-onset progressive HL. As for USH1B cases, all patients showed congenital or early-onset severe-to-profound HL.

**Figure 2:**
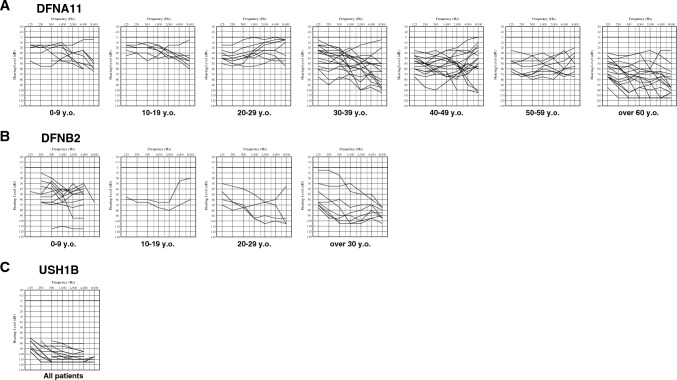
Overlapping audiograms for each age group for each hereditary form. (**A**) Overlapping audiograms for each age group among DFNA11 patients showed progressive HL that gradually worsened from mild-to-moderate and high-frequency sloping HL to flat-type severe-to-profound HL. For (**B**) DFNB2 cases, their overlapping audiograms also showed progressive HL and about half of the cases over 30 years of age showed severe-to-profound HL. (**C**) USH1B patients showed congenital severe-to-profound HL.

For DFNA11, most of the cases in this study showed late-onset progressive HL. About the half of the DFNA11 patients developed or became aware of their HL in their first or second decade; however, about half of the cases experienced HL onset after their second decade (Fig. 1A). Most of the ADNSHL patients showed mild-to-moderate and high-frequency sloping HL. Overlapping audiograms for each age group among the ADNSHL patients showed progressive HL that gradually worsened to flat-type severe-to-profound HL (Fig. 2A). In terms of development, there were no delays observed for the average month at which walking started and at which the neck was supported when sitting (4.11 months and 12.5 months, respectively).

With regard to DFNB2 cases, almost all cases showed congenital or early-onset progressive HL. The onset of HL in DFNB2 cases was in their first decade (Fig. 1B). The severity and audiometric configuration for DFNB2 varied among patients. Overlapping audiograms for each age group among the ADNSHL patients showed progressive HL, and about half of the cases over 30 years of age showed severe-to-profound HL (Fig. 2B). For DFNB2 patients, no developmental delays were observed for the average month at which walking started and at which the neck was supported when sitting (3.62 months and 13.2 months, respectively). Families #12 and #17 showed an AD family history. Patient #12 who carried p.E513Q and p.R1977Q variants, was a 56-year-old female. Her mother is 84 years old and may suffer age-related hearing loss. Unfortunately, we could not obtain detailed audiograms for the mother. However, the p.E513Q variant was identified from four other sporadic cases (family #9, #10, #11 and #13) in combination with several variants. In addition, p.R1977Q was previously reported as a genetic cause of USH1B. Thus, we concluded this case to be DFNB11. Another case was observed in family #17, where a 38-year-old male carried a homozygous p.G1159S variant. From an interview with the proband, his father also had hearing loss, but his father has already died and we could not obtain detailed information. However, a p. G1159S variant was identified from three other autosomal recessive families and three sporadic cases (family #2, #3, #15, #16, #18 and #19). From this result, we concluded that this patient was also DFNB11.

For USH1B cases, all patients showed congenital or early-onset severe-to-profound HL (Figs. 1C and 2C). All patients aged 10 years or older were diagnosed with RP and/or complained of visual symptoms including night blindness, narrowing of visual field or both. Some patients developed visual symptoms in their first decade. One case was identified with USH1B prior to the onset of visual symptoms based on the results of genetic testing. It is noteworthy that one ARNSHL patient (#19) showed relatively late-onset RP (onset at 22 years old). However, another patient with the identical combination of variants (#20) has not been diagnosed with RP or complained of any visual symptoms at age 38. Thus, the RP observed in case #19 may have been the result of other causes. In terms of development, the average month at which the neck was supported when sitting was normal at 4.85 months, but that of the month at which walking started was significantly delayed at 20.5 months.

### Genotype–Phenotype correlations observed in this study

All variants identified from ADNSHL patients were missense or in-frame deletion variants, and no null variants (nonsense, splicing and frameshift variants) were observed (Tables 1 and 3). In ARNSHL or USH1B patients, both missense variants and null variants were identified (Tables 2, 4 and 5). As shown in Table 4, the combination of the identified variants for all ARNSHL patients carried at least one missense variant, and there were no cases with biallelic null variants. On the other hand, almost half of the patients with Usher syndrome carried biallelic null variants (Table 5).

Almost half of the missense variants for ADNSHL and ARNSHL were located in the Myosin head domain, with the few exceptions located in MyTH4 domain, SH3 domain or FERM domain. On the other hand, the majority of missense variants in USH1B families were located in the posterior half region including the MyTH4 domain, Band4.1 domain and FERM domain. Thus, we estimated that the HL caused by variants located in later half tended to be more severe than that caused by the variants located in the Myosin head domain.

### Intervention for HL and outcomes

**Figure 3:**
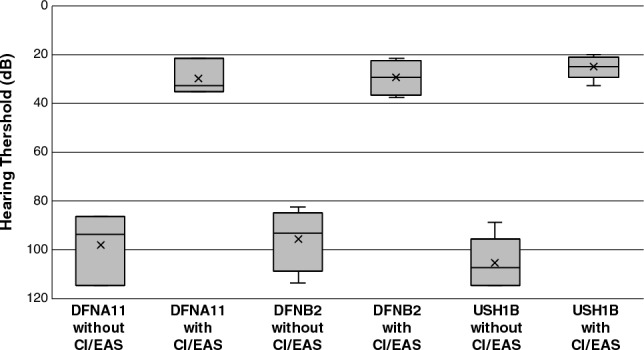
Hearing threshold of patients with/without CI/EAS for each hereditary form. DFNA11, DFNB2, and USH1B. CI/EAS showed good outcomes for patients with *MYO7A* variants. The corresponding two groups were tested by t test.

Most of the ADNSHL or ARNSHL patients with mild-to-severe HL used hearing aids, although some received cochlear implants. On the other hand, almost all Usher syndrome patients showed severe-to-profound HL and most of them received cochlear implants in childhood. The outcome of cochlear implantation (CI) was favorable (Fig. 3), indicating that CI affords a good treatment option for the patients with severe-profound *MYO7A*-associated HL in all hereditary forms.

## Discussion

In this study, we showed the prevalence of *MYO7A*-associated HL in a large HL cohort was 1.36% (137/10,047). The prevalence of *MYO7A*-assocciated ADNSHL in autosomal dominant or maternal inheritance HL patients was 4.06% (91/2,243), while it was 0.38% (25/6,163) for ARNSHL patients and 0.32% (21/6,163) for *MYO7A*-associated Usher syndrome cases among autosomal recessive or sporadic HL patients. This is the first paper reporting the prevalence of all three clinical phenotypes of *MYO7A*-associated HL identified from a single large cohort. Most previous papers reported only on Usher syndrome cases or a limited number of ADNSHL or ARNSHL patients, so the prevalence as well as the detailed clinical characteristics for ADNSHL and ARNSHL *MYO7A*-associated HL has been unclear. In previous papers, Sloan-Heggen et al., reported the genetic analysis results for 1119 hearing loss patients (Caucasian, Hispanic, African American, Asian, Middle Eastern, Ashkenazi Jewish and others), with the prevalence of *MYO7A*-associated HL being 1.79% (20/1119 cases)^12^. Among the 20 cases, one case was DFNA11 (0.7%, 1/141 autosomal dominant HL cases), three cases were DFNB2 (0.36%, 3/830 autosomal recessive HL or sporadic cases), and 16 cases were USH1B (1.68%, 14/830 autosomal recessive HL or sporadic cases, one case identified as AD, and one case identified with an unknown family history). Ma et al.^13^, reported the NGS analysis results for 879 Chinese HL patients, with the prevalence of *MYO7A*-associated HL being 2.39% (21/879 cases). Eleven cases were DFNA11 (6.88%, 11/160 autosomal dominant HL cases) and 10 cases were DFNB11 or USH1B (1.70%, 10/589 autosomal recessive HL or sporadic cases). Abu Rayyan et al.^14^, reported NGS analysis results for 491 Palestinian families with HL, and identified 28 *MYO7A*-associated HL cases (5.7%, 28/491). All 28 cases in their report were DFNB11 or USH1B. Baux et al.^15^, reported the NGS analysis results for 207 French hearing-impaired patients, and identified 5 cases with *MYO7A*-associated HL (2.4%, 5/207). The prevalence of *MYO7A*-associated HL in previous reports varied depending on the sample number and clinical characteristics of the cohort, ranging from 1.79 to 5.7%. The prevalence of *MYO7A*-associated HL in this study was 1.36%, which was similar to those in previous reports. There are several papers reporting NGS analysis results for hearing loss patients; however, many of them pre-screened *GJB2-* or *SLC26A4*-associated HL cases, making it difficult to estimate the true prevalence of *MYO7A*-associated HL. Our results will shed light on the detailed clinical features of HL, especially for the *MYO7A*-associated ADNSHL and ARNSHL patients.

Interestingly, some of the identified variants from ADNSHL patients in this study were commonly observed even in different families. This result suggested that a common founder mutation or mutational hot spots are associated with this multi-familial identification. The fact that most of the commonly identified variants in this study are only identified from Japanese HL patients or from Japanese and East Asian HL patients supports the notion that these variants were founder mutations.

As for the clinical characteristics of ADNSHL patients, all patients showed delayed onset HL that usually developed after language acquisition (post-lingual onset). In addition, we clearly observed progressive high-frequency HL in a large number of patients. These clinical characteristics were consistent with previous reports^16^, but the patient number in this study is the largest to date and these findings will be useful to our understanding of the clinical characteristics of *MYO7A*-associated ADNSHL. Most of the DFNA2 variants identified in this study were novel, with few previously reported. In a previous study, Sang et al., reported a DFNA11 family with a c.2003G>A variant^17^. The clinical characteristics for this family were mild-to-severe progressive HL with an onset age of 17–45 years. The clinical characteristics for DFNA2 cases in this previous report were consistent with those in our cases.

Congenital or early-onset HL was observed in patients with *MYO7A*-associated ARNSHL. Most of the USH1B cases showed congenital severe-to-profound HL and first- or second-decade onset RP, which is consistent with previous reports^18^. Unlike DFNA11 variants, many of the DFNB2 and USH1B variants identified in this study were previously reported. The clinical phenotypes for these autosomal recessive cases were dependent on the combination of variants. In previous reports, most DFNB2 cases showed first decade onset severe-to-profound HL^19,20^, and most of USH1B cases showed congenital severe-to-profound HL, with RP diagnosed in the first-to-second decade^21–23^.

With regard to genotype/phenotype correlations, all DFNA11 patients carried non-truncating variants (missense or in-frame deletion), and all DFNB2 patients carried at least one non-truncating variants with both the truncating or non-truncating variant in the *trans* configuration. On the other hand, about half of the USH1B patients carried biallelic truncating variants. These results suggested that the pathogenic mechanism for *MYO7A*-associated ADNSHL might be a dominant negative effect, whereas the pathogenic mechanism for *MYO7A*-associated ARNSHL and USH1B might be a loss of function. Further, the residual function of each *MYO7A* variant is thought to be associated with the phenotypic differences in ARNSHL and USH1B.

A similar situation was reported for *CDH23*-associated HL, which is caused by ARNSHL (DFNB12) or Usher syndrome (USH1D). The DFNB12 phenotype is reported to be associated with biallelic missense mutations, whereas the USH1D phenotype is associated with presumably functional null alleles, including nonsense, splicing, frameshift, or some missense mutations^24^. In addition, it has been reported that patients with compound heterozygous variants of USH1D and DFNB12 show a non-syndromic phenotype^25^.

It is difficult to distinguish between ARNSHL and USH1B, particularly in younger patients, as RP in USH1B patients develops between age 5–15 years and it is difficult to identify RP in younger patients without any visual symptoms. Therefore, it is impossible to strictly distinguish ARNSHL patients from USH1B patients. Thus, some of ARNSHL patients identified in this study may develop visual symptoms later. Of course, follow-up is important for the identification of visual symptoms even in ARNSHL cases. In this study, we collected data for “the month at which the head is supported when sitting” and “the month at which walking started” as indirect evidence with which to elucidate the vestibular dysfunction that characterizes USH1B. Most of the USH1B cases showed delays in balance when sitting and/or walking but there were few cases with such delays among the ADNSHL and ARNSHL patients. Furthermore, the average month at which walking started was delayed in USH1B cases. Loundon et al., reported the average age at which walking starts for USH1B cases was 20 months, suggesting vestibular problems. Our results for the start of walking for USH1B cases showed a significant delayed at 20.5 months, which is consistent with the previous report^26^.

Additionally, we also identified another genotype–phenotype correlation in this study. Almost half of the missense variants in ADNSHL and ARNSHL patients were located in the Myosin head domain, with few located in the MyTH4 domain, SH3 domain or FERM domain. On the other hand, the majority of missense variants in the USH1B families were located in the posterior half region including the MyTH4 domain, Band4.1 domain and FERM domain. Joo et al.^27^, reported that *MYO7A*-asssociated ADNSHL patients with Myosin head domain variants showed relatively milder HL than did patients with variants in the MyTH4 domain, which appears to support our findings.

In conclusion, next-generation sequencing analysis successfully identified 34 previously reported variants and 36 novel variants in *MYO7A*-associated HL patients. The estimated prevalence of *MYO7A*-associated hearing loss in the Japanese hearing loss cohort was 1.36% for all patients, 4.06% for ADNSHL among autosomal dominant or maternal inheritance cases, 0.38% for ARNSHL and 0.32% for USH1B among autosomal recessive or sporadic hearing loss cases. This large cohort study of hearing loss patients provided valuable new insights, particularly with regard to hearing deterioration in *MYO7A*-associated ADNSHL patients. This information is expected to be useful for the provision of more appropriate intervention for *MYO7A*-associated HL patients. In addition, understanding the gene involved in hearing loss also opens up possibilities for new future therapies (such as gene therapy).

## Materials and methods

### Subjects

A total of 10,042 HL patients from 102 institutions across Japan participated in this study. Clinical information and peripheral blood samples were obtained from patients and their relatives. Written informed consent was obtained from all patients (or from their next of kin, caretaker, or legal guardian in the cases of minors or children) and relatives. This study was approved by the Shinshu University Ethical Committee, as well as the respective ethical committees of the other participating institutions, and was conducted in accordance with the Declaration of Helsinki. The estimated inheritance pattern was classified into “autosomal dominant or maternal inheritance” with two or more generations of family members suffering hearing loss, “autosomal recessive” with two or more siblings suffering hearing loss, and “sporadic” in cases without any affected family members.

### Next-generation sequencing and bioinformatic analysis

Next-generation DNA sequencing was performed for the 63 target genes reported to cause non-syndromic hearing loss as described in a previous report^28^. In brief, amplicon libraries were prepared using the Ion AmpliSeq Custom Panel, with the Ion AmpliSeq Library Kit 2.0 and the Ion Xpres Barcode Adapter 1-96 Kit (ThermoFisher Scientific) according to the manufacturer’s instructions. After amplicon library preparation, emulsion PCR, and next-generation sequencing were performed with an Ion 200 sequencing kit (ThermoFisher Scientific) and Ion PGM sequencer (ThermoFisher Scientific) or an Ion HiQ chef Kit (ThermoFisher Scientific) and Ion Proton sequencer (ThermoFisher Scientific) according to the manufacturer’s protocol. The sequence data were mapped against reference human genome sequence (build GRCh37/hg19) with the Torrent Mapping Alignment Program (TMAP). The DNA variants were detected with the Torrent Variant Caller plug-in software (ThermoFisher Scientific). Copy number variation analysis was also performed for all patients by using read depth data according to the copy number variation detection methods described in our previous report^29^; however, no copy number variations for the *MYO7A* gene were identified.

After variant detection, annotation of identified variants was performed with ANNOVAR software. The missense, nonsense, insertion, deletion, and splicing variants were selected from among the identified variants. For the variants located in the exon–intron border region (including synonymous variants and intronic variants), candidate variants predicted to affect splicing by in silico splicing prediction dbscSNV^30^ were also selected.

Variants were further selected as less than 1% of several control population database including the 1000 genome database^31^, the 6500 exome variants^32^, The Genome Aggregation Database^33^, the 1200 Japanese exome data in Human genetic variation database^34^, the 38,000 Japanese genome variation database^35^ and the 333 in-house Japanese normal hearing controls. All filtering procedures were performed using original database software described previously^36^.

The pathogenicity of identified variants was analyzed in accordance with the American College of Medical Genetics (ACMG) standards and guidelines^37^ with the ClinGen hearing loss clinical domain working group expert specification^38^. Variants were defined as candidate variants if the following criteria was fulfilled; (1) for the variants previously reported as “pathogenic” or “likely pathogenic” without any contradictory evidence, we applied the same pathogenicity classification, (2) novel variants classified as “pathogenic” or “likely pathogenic” were considered as strong candidates for each case, (3) variants of “uncertain significance” (VUS) identified as only one candidate after the filtering procedure without any candidate variants in the other 62 genes were also included, (4) two variants found in recessive inheritance cases, and (5) there was no contradiction with the family analysis.

We performed Sanger sequencing analysis to validate the identified variants according to the manufacturer’s instructions. All PCR and sequencing primers were designed using the web version Primer 3 plus software^39^.

### Clinical evaluation

Clinical information, including sex, onset of HL, age and hearing thresholds at genetic testing, episodes of tinnitus and vertigo, progression of HL, type of interventions: hearing aid (HA) or CI was collected from a review of medical charts. For the subjects with ARNSHL or Usher syndrome, we also collected the age of RP diagnosis, onset of visual symptoms, months at which walking started, months at which the neck was supported when sitting and the results of caloric testing, if available. Evaluation of HL was performed by pure-tone audiometry. The pure-tone average (PTA) was calculated from the audiometric thresholds at four frequencies (500, 1000, 2000, and 4000 Hz). The severity of HL was classified into five categories: normal (PTA under 20 dB), mild (PTA 20–40 dB), moderate (PTA 41-70 dB), severe (PTA 71–90 dB), and profound (PTA > 91 dB). The audiometric configurations were categorized into Flat, Low-frequency ascending, Mid-frequency U-shaped, High-frequency gently sloping, and High-frequency steeply sloping, as reported previously^40^. If the patients couldn’t have pure-tone audiometry due to their age (approximately aged four or under) or other reasons, the auditory steady-state response (ASSR), conditioned orientation response audiometry (COR) or play audiometry were performed. The outcome of interventions (HA or CI) was evaluated by PTA.

### Supplementary Information

Supplementary Figures.

## Data Availability

The datasets used during the current study are available from the corresponding author on reasonable request.
