# Supplementary material for: The prevalence and clinical features of MYO7A-related hearing loss including DFNA11, DFNB2 and USH1B
Source: Sci Rep. 2024 Apr 9;14:8326. doi: 10.1038/s41598-024-57415-1 (PMC11003999; doi:10.1038/s41598-024-57415-1)

Supplemental Figure 1 Family segregation analysis results for DFNA11 cases

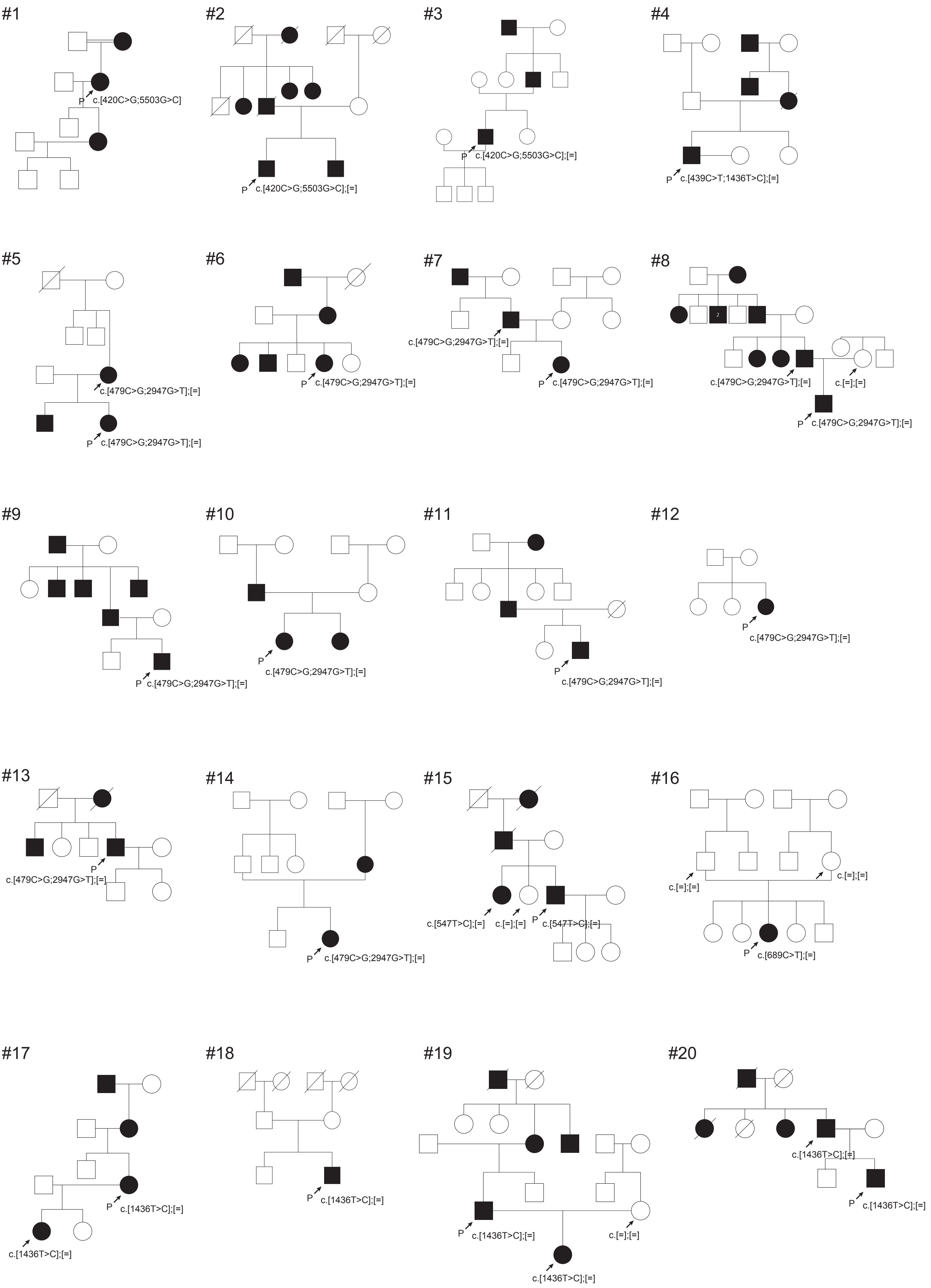

Supplemental Figure 1 continued

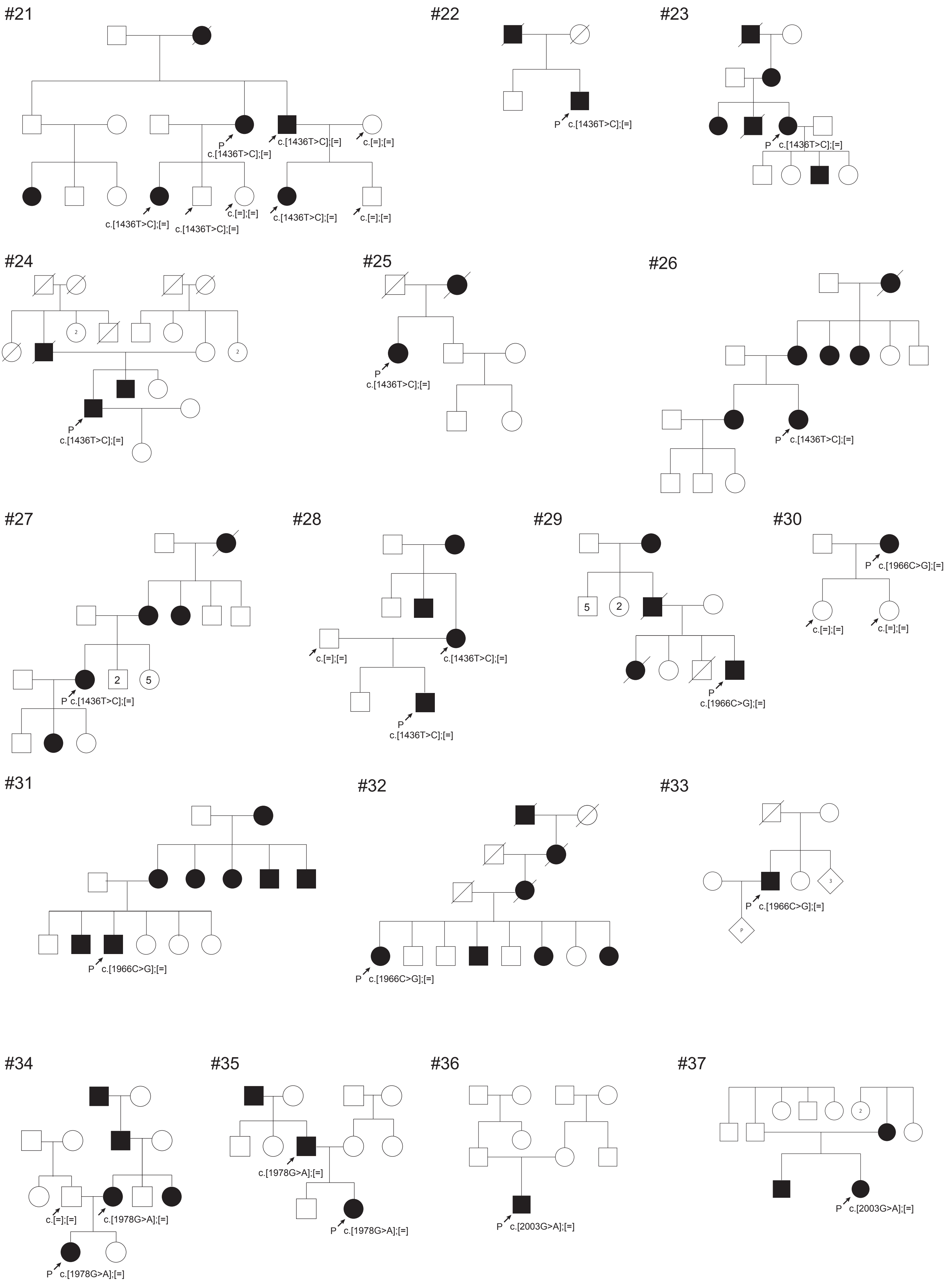

Supplemental Figure 1 continued

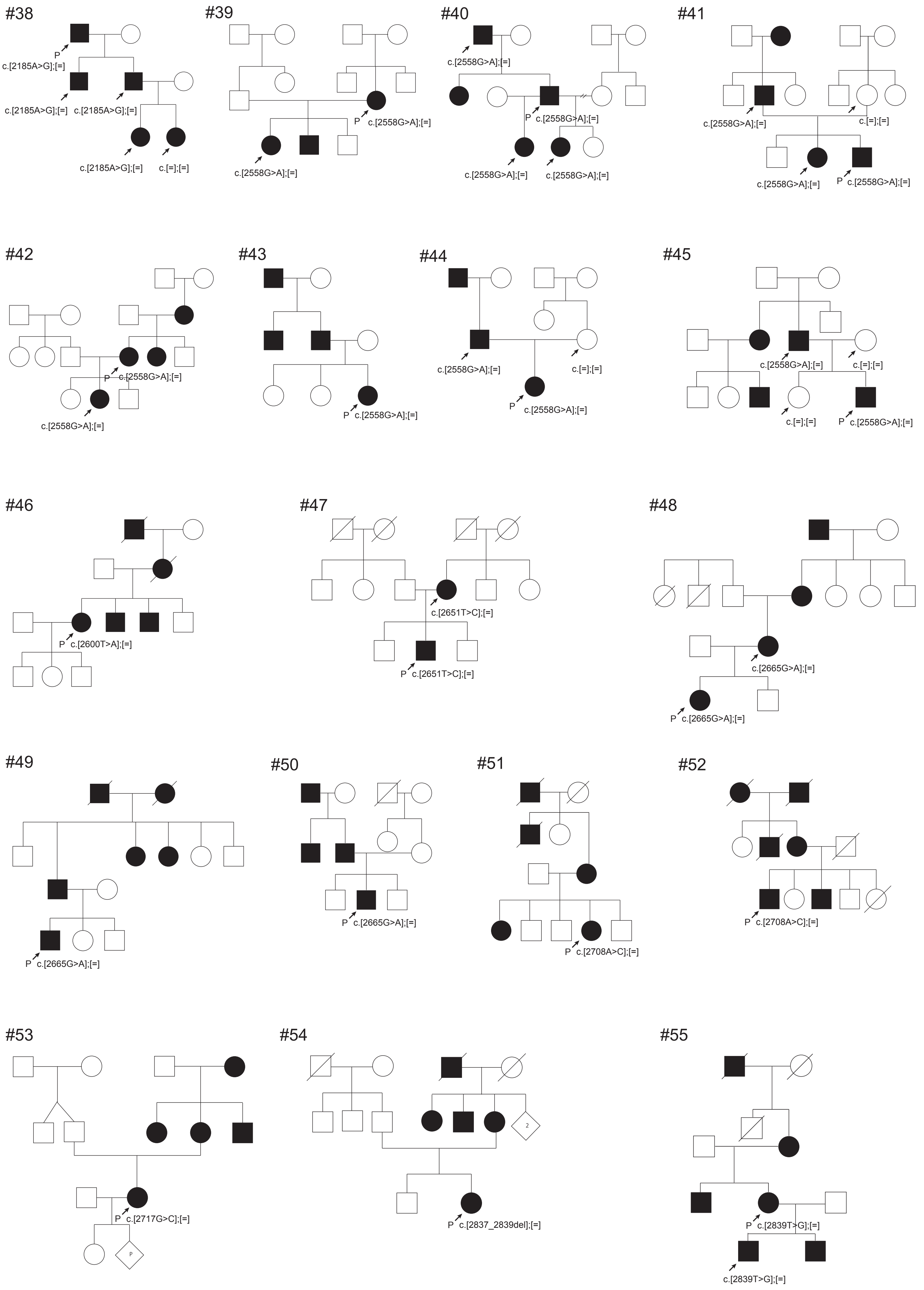

Supplemental Figure 1 continued

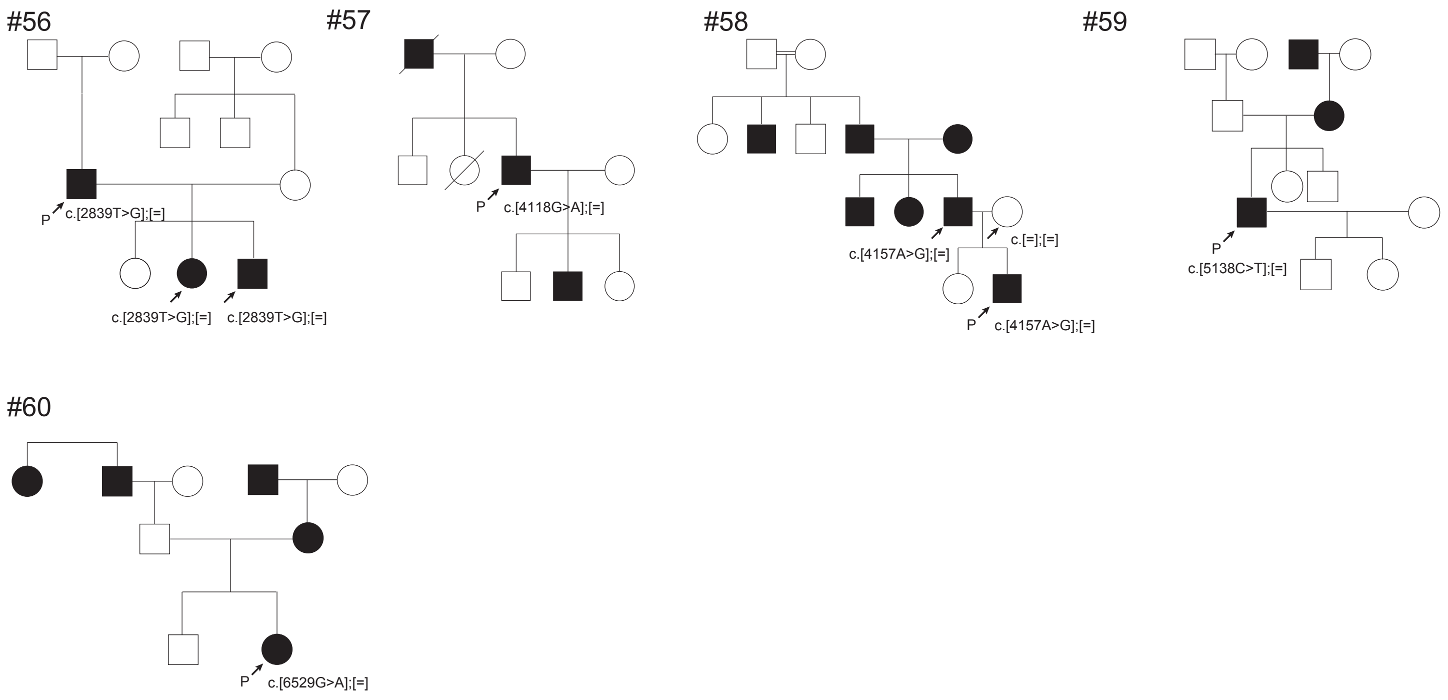

Supplemental Figure 2 Family segregation analysis results for DFNB2 cases

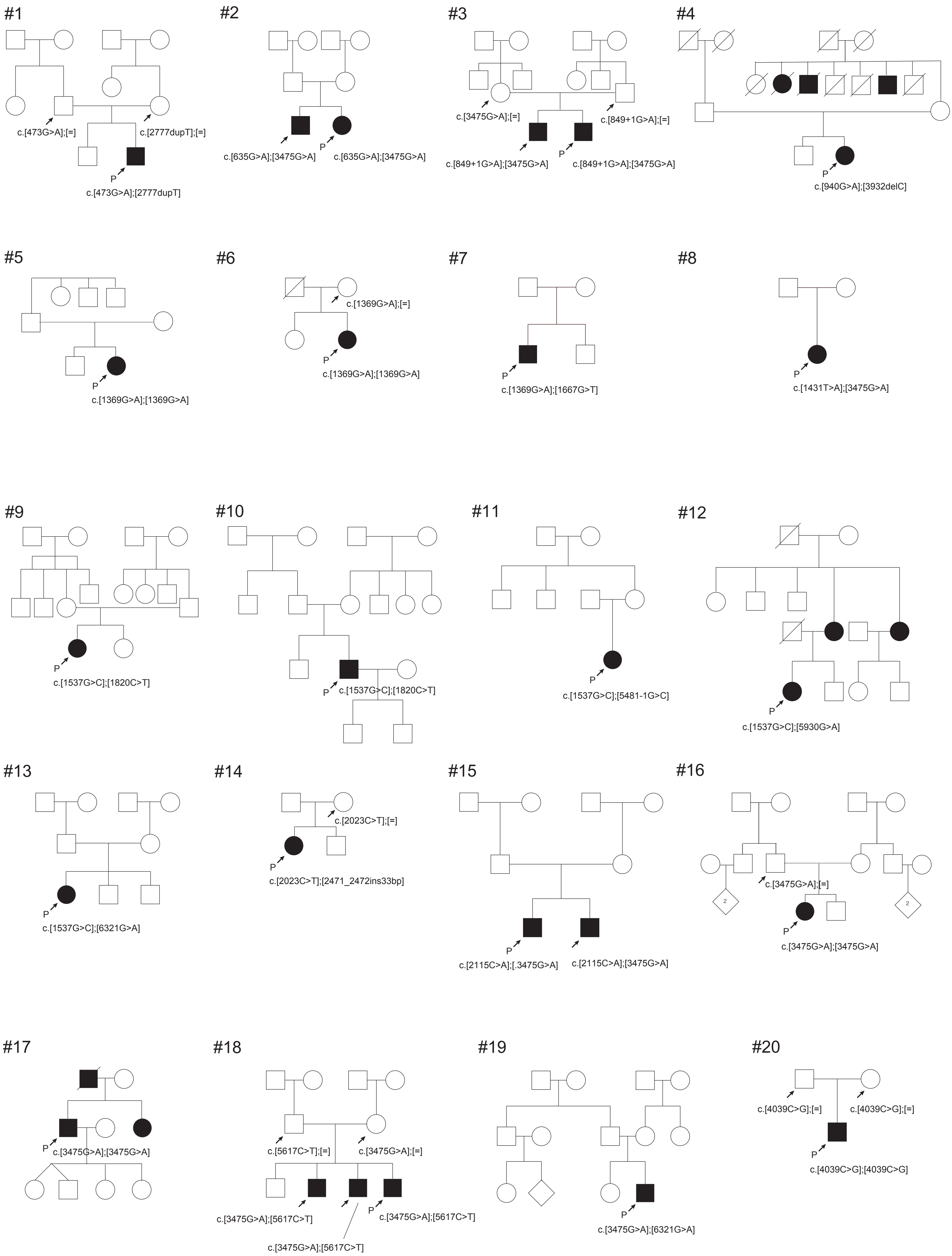

Supplemental Figure 3 Family segregation analysis results for USH1B cases

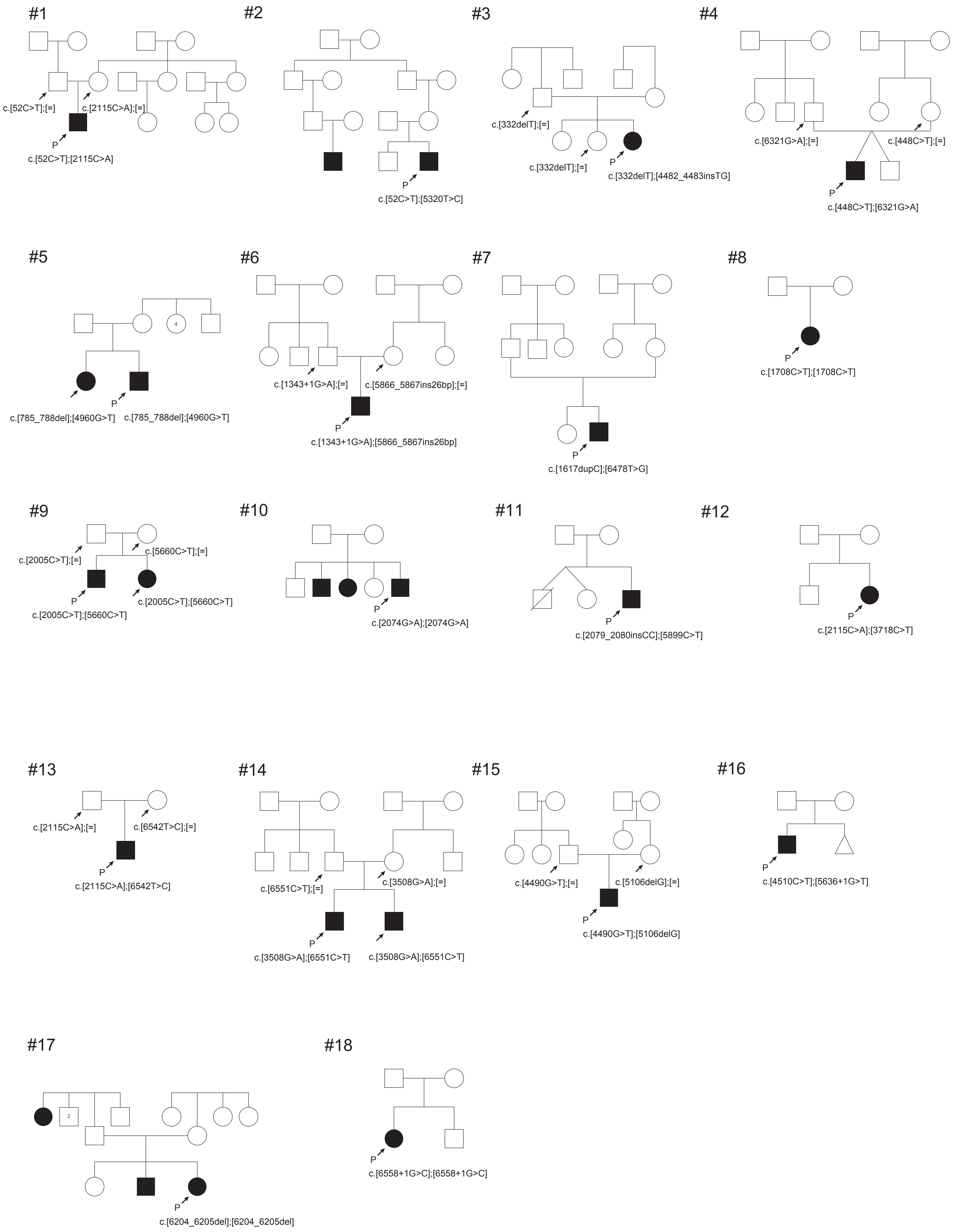

Supplement: Supplementary file 1 — Supplementary Figures. [file 41598_2024_57415_MOESM1_ESM.pdf]
